# Reparation, rights, and dissidence: a qualitative study of feminist advocacy for legal, safe, and free abortion in Ecuador

**DOI:** 10.1080/26410397.2026.2698235

**Published:** 2026-07-08

**Authors:** Cynthia Carofilis, Paz Guarderas, Isabel Goicolea

**Affiliations:** a Researcher, Psychosocial Research Group, Universidad Politécnica Salesiana, Quito, Ecuador.; c Professor, Department of Epidemiology and Global Health, Umeå University, Umeå, Sweden

**Keywords:** abortion, feminist advocacy, sexual and reproductive rights, Ecuador, tactical strategy

## Abstract

This article investigates the political practices related to abortion in Ecuador from their politicisation in 2006 until 2023, two years after the decriminalisation of abortion in all rape cases. We identify: (1) the discursive and embodied practices that construct and politicise the aborting body within feminist advocacy for legal, safe, and free abortion in Ecuador; (2) the effects of these practices; and (3) feminist challenges to conservative discourses that, in some cases, appropriate their rhetorical strategies. Based on participant observations of feminist demonstrations and the analysis of texts produced by Ecuadorian feminist organisations, we trace three “tactical strategies” (using Lugones’ conceptualisation) that introduce different configurations of the body and propose different effects: (1) abortion as reparation, along with the notion of the victim body; (2) abortion as a right, along with the citizen body; and (3) abortion as dissidence, along with the configuration of the sovereign body. These tactical strategies need to be understood within the broader political context and evolving anti-choice forces. The coexistence of different strategies can lead to both complementary effects and friction.

## Plain language summary

In this study, we explored how feminist collectives advocated for legal, safe, and free abortion in Ecuador from 2006 until 2023. These two years followed the decriminalisation of abortion in all rape cases. We used participatory observation and document analysis. We identified overall “tactical strategies” that activists are using. The concept of “tactical strategies” was developed by Latin American feminist researcher Maria Lugones to bring together both high-level strategies and the everyday practices (tactics) that social groups use to achieve their aims. The concept criticises the divide between “grand” strategies and “minor” tactics. Everyday acts of resistance are seen as just as important and relevant as better known actions, such as political advocacy. Through the analysis, we identified three tactical strategies: reparation, rights and dissidence. Reparation foregrounds a girl who is pregnant as a consequence of rape or incest. She is portrayed as a victim in need of an abortion as a form of repairing the violence. Rights are associated with the woman citizen who can exercise her right to health with the support of the legal and medical system. Dissidence does not trust official or institutional structures to ensure access to safe, legal, and free abortion. It centres around autonomous networks of feminist accompaniment for self-managed abortion. Each of these tactical strategies poses possibilities and challenges. How they are used depends on the social and political contexts. Sometimes they complement each other, but at other times, they may be contradictory.

## Résumé

Cet article examine les pratiques politiques liées à l’avortement en Équateur, de leur politisation en 2006 jusqu’en 2023, soit deux ans après la dépénalisation de l’avortement dans tous les cas de viol. Nous identifions : (1) les pratiques discursives et incarnées qui construisent et politisent le corps qui avorte dans le cadre du plaidoyer féministe pour un avortement légal, sûr et gratuit en Équateur ; (2) les conséquences de ces pratiques ; et (3) les contestations féministes des discours conservateurs qui, dans certains cas, s’approprient leurs stratégies rhétoriques. À partir de l’observation des participants de manifestations féministes et de l’analyse de textes produits par des organisations féministes équatoriennes, nous retraçons trois stratégies tactiques qui présentent différentes configurations du corps et proposent différents effets : (1) la stratégie tactique de l’avortement comme réparation, associée à la notion du corps victime ; (2) la stratégie tactique des droits, érigés en lien avec le corps citoyen ; et (3) la stratégie tactique de la dissidence, associée à la configuration du corps souverain. Ces stratégies tactiques doivent être comprises dans le contexte politique plus large et face à l’évolution des forces anti-avortement. La coexistence de différentes stratégies peut engendrer à la fois des effets complémentaires et des frictions.

## Resumen

Este artículo investiga las prácticas políticas relacionadas con el aborto en Ecuador desde su politización en 2006 hasta 2023, dos años después de la despenalización del aborto en todos los casos de violación. Identificamos: (1) las prácticas discursivas y corporizadas que construyen y politizan el cuerpo que aborta en el marco de incidencia política feminista a favor del aborto legal, seguro y libre en Ecuador; (2) los efectos de esas prácticas; y (3) cómo estas prácticas feministas desafían los discursos conservadores y en ciertos casos se apropian de sus estrategias retóricas. A raíz de observaciones participantes de manifestaciones feministas y del análisis de textos producidos por organizaciones feministas ecuatorianas, seguimos tres estrategias tácticas que presentan diferentes configuraciones del cuerpo y proponen diferentes efectos: (1) la estrategia táctica del aborto como reparación, junto con la noción del cuerpo víctima; (2) la estrategia táctica de derechos, que son erigidos junto con el cuerpo ciudadano; y (3) la estrategia táctica de disidencia, junto con la configuración del cuerpo soberano. Estas estrategias tácticas deben entenderse dentro del contexto político general y de fuerzas evolutivas anti-aborto. La coexistencia de diferentes estrategias puede propiciar tanto efectos complementarios como fricción.

## Introduction

Feminist struggles for the decriminalisation of abortion in Ecuador have unfolded through diverse strategies and actors, in a process marked by both advances and setbacks. After 1938, the Ecuadorian Criminal Code permitted abortion under only two circumstances: when the life of the mother or fetus was at risk, and in cases of rape involving women with disabilities – a narrow reform that marked the country’s first legal departure from total prohibition of abortion. This restrictive framework has gradually been challenged and reshaped by feminist and women’s movements in a non-linear process. In the early 2000s, in the aftermath of transnational meetings promoting sexual and reproductive rights, their efforts focused on securing access to comprehensive sex education and contraceptives, including emergency contraception.^1^ From 2009 onwards, information hotlines for self-managed abortion began to operate, expanding access to knowledge and support under the premise of the right to information. Organisations such as the *Coordinadora Política de Jóvenes* (Youth Political Coordination) and the *Coalición por la Descriminalización del Aborto* (Coalition for Abortion Decriminalisation) sought to enshrine the issue in the debates for drafting the 2008 Ecuadorian Constitution, without success. In 2013, an amendment to the *Código Orgánico Integral Penal* (COIP, Organic Comprehensive Criminal Code) explicitly restricted abortion to only three exceptions, while criminalising all other cases, resulting in the prosecution of hundreds of women.^2^ In response, feminist accompaniment networks emerged offering legal and psychological support, while advocacy efforts intensified. In 2015, there were further restrictions on sexual rights, and conservative discourses in the public sphere became more extreme.^3,4^ During this time, feminist groups sought to expand abortion rights through a bill that won majority support but was ultimately vetoed by President Lenín Moreno. In response, organisations challenged Article 150 of the COIP (which only allowed the termination of pregnancy for women who had been raped if they also suffered from mental disabilities) through campaigns supported by transnational networks, and energised by the global Green Tide movement. This movement, which originated in Argentina, is symbolised by a triangular green scarf inspired by the white scarves worn by the Mothers of Plaza de Mayo protesting the military dictatorship. Between 2019 and 2021, the Constitutional Court consolidated seven lawsuits on abortion into Case 34–19-IN/21, marking a turning point. Following regional advances in Argentina, Mexico, and Colombia, Ecuador made some progress in 2022 when sustained feminist pressure led to the decriminalisation of abortion for all women in cases of rape.

Feminist mobilisation has not stopped there (Table 1), and Ecuadorian feminists continue to take to the streets and strive to influence decision-makers to expand abortion rights. This is an uphill struggle in which feminists in Ecuador, as is the case elsewhere, are facing the power of the anti-rights movement, which is extremely influential within the different branches of the state.^1^ Far from being unique, the process in Ecuador mirrors what has happened in other parts of Latin America.

**Table 1.** Timeline of debates around abortion (2004-2025), focusing on key milestones in Ecuador, Latin America, and the United States

2004	**Ecuador:** Debates on the commercialisation of emergency contraception. Conservatives demand its ban.
2005	National campaign for the right to legal, safe, and free abortion in **Argentina**.
2008	**Ecuador**: Debate around bodily autonomy in the National Constituent Assembly. The Women's Health Collective launches the first abortion hotline in **Latin America**.
2012	**Uruguay** becomes the second country in Latin America to decriminalise abortion during the first trimester. The **Inter-American Court of Human Rights** establishes the distinction between conception and in vitro or intracorporeal fertilisation.
2013	The **Ecuadorian** National Assembly debates an amendment to the Comprehensive Organic Criminal Code (COIP in Spanish).
2015	The Ni Una Menos March sweeps through **Argentina, Mexico, Uruguay, Ecuador**, and **Colombia**, uniting voices on the street against gendered violence. In **Ecuador**, the Guide for Therapeutic Abortion is approved.
2016	International Women´s Strike
2018	A bill to decriminalise and legalise abortion is debated in the Argentinian Congress. Green Tide in **Argentina** paves the way as a national protest for abortion rights.
2019	Green Tide spreads in **Latin America**. **Ecuador**: National Assembly debates the decriminalisation of abortion in cases of rape.
2020	**Argentina**: Passes the “Access to Voluntary Interruption of Pregnancy” Law
2021	The **Ecuador** Constitutional Court rules in favour of passing a law in Ecuador to decriminalise abortion in all cases of rape. The Supreme Court in **Mexico** rules that penalising abortion is unconstitutional.
2022	**Colombia**'s Constitutional Court legalises abortion up to 24 weeks of pregnancy. **United States**: Supreme Court overturns Rode vs Wade, ending the constitutional right to an abortion.
2024	Feminist movements in **Ecuador** petition the Constitutional Court to repeal Article 149 of the COIP, which criminalises abortion.
2025	**United States** cuts USAID programmes and reinstates the Global Gag Rule, prohibiting support for abortion and other sexual and reproductive rights programmes worldwide.

Note: Elaborated by authors.

As the timeline depicts, progress is accompanied by backlash, and national and transnational collectives advocating for abortion rights need to adapt to changing scenarios and innovate to counterbalance the rise of anti-choice movements. In this paper, we analyse the different tactical strategies mobilised by feminist movements in Ecuador in their fight for legal, safe, and free abortion. In the current context, characterised by the increasing influence of anti-choice movements,^5^ it is more urgent than ever to understand and learn how feminists mobilise and adapt their strategies to changing national and global scenarios.

### Tactical strategies: conceptual and analytical approach

For this analysis, we used as a starting point the concept of tactical strategies proposed by María Lugones.^6^ Lugones questioned the distinction between strategy and tactic, because this distinction represents strategy as superior to tactics and the only position from which to theorise. Lugones argued that tactics are represented as inferior not only because they focus on everyday struggles, and they are understood as unanalytical, but also because they have the potential to be deviant, abnormal, or even criminal. In the specific field of activism for safe and free abortion, a strategy can be, for example, lobbying with politicians to change laws that criminalise abortion; while tactics relate to the way women started taking misoprostol pills for self-managed abortions in Brazil, and how this information was spread through informal networks. Lugones questioned this hierarchising dichotomy and proposed the alternative concept of “tactical strategies”. Unlike the concept of strategies, which Lugones criticises for being disengaged, tactical strategies remain “within the concreteness of body-to-body engagement” as they pay embodied attention to the micro-mechanisms of power and how they are being met with creative resistance. The history of abortion activism shows how the concept of tactical strategies is a more productive way to explain how some tactics that may seem mundane, individualistic and reaching few (like taking misoprostol pills) could become a global movement (for example, accompaniment networks and organisations that facilitate the distribution of abortion pills across borders), and how grand strategies, like decriminalisation through political lobbying, may, in fact, not solve all access problems.

For Lugones, everyday struggles and large-scale political lobbying are not two separate spaces, and neither of them has greater value or interest than the other; rather, theorising stems from the practice. Lugones draws upon Michel De Certeau’s^7^ distinction between the strategic and the tactical. The strategist “sees” from a point of view characterised by the distance of height and abstraction. In contrast, the tactical strategist is capable of recognising oppressive discourses and how they emerge in concrete practices, which entails a specific form of perceiving reality and engaging in it.

Tactical strategies refer to practices of resistance to intermeshed oppressions, that incorporate bodies and are handled in the interstices of ideology. Lugones emphasises how these practices disrupt the dichotomy between theory and practice, the discursive and the embodied. This concept makes it possible to analyse the capacity to theorise resistance from the position of “subaltern” movements and the potentiality of the different spaces within which resistance to hegemonic anti-rights discourses is generated.

### An overview of feminist mobilisation for legal, safe, and free abortion in Latin America

Studies in the Latin American region have pointed out the multiplicity of strategies used by feminist movements. Some of the literature analyses these strategies as discursive frames. Sutton and Borland^8^ analysed the written conclusions of the *Encuentros* (national feminist meetings in Argentina). They found multiple frames, with public health, economic and social justice, and choice being the most frequently deployed. Ruibal and Fernández Anderson,^9^ investigating the Argentinian case, found three main strands of strategies: legal reform, ensuring access to abortions that are already available within the existing legal frames, and direct action. The authors connect these strategies to transnational initiatives throughout Latin America. The first strand finds concrete expression in the “September 28th Campaign for the Decriminalisation of Abortion in Latin America and the Caribbean,” a broad regional network of abortion reform active since 1990. The second strand has been developed within a regional coalition (*Causal Salud* – health exception), and the third is connected to the development of hotlines across the region. Other scholars have pointed out the importance of transnational networks extending beyond nation states for advancing abortion rights in the Latin American region. Ferreto^10^ described a “regionalizing” effect leveraged through networks and alliances that promote a structure of opportunities complementary to those of national states. Cyber-feminist activism has constituted a massive and intersectional collective in Latin America, linking current feminist activism with past struggles and resistance movements.^11^ Other academic work connects political struggles for the right to abortion with the expansion of democracy, particularly around the Green Wave. Most articles analyse the Green Wave as a Latin American movement.^12–14^

Some of the strategies analysed build upon the concept of bodily autonomy and the feminist political practice of the body-territory,^15,16^ such as access to self-managed abortion through networks of abortion accompaniment.^15,17^ In addition, occupying public spaces through performances and emotive rituals is part of so-called “cultural activism”.^18^ Symbols, frames, and discourses travel to connect the struggles for abortion in Latin America and worldwide. Nayla Vacarezza’s work^14^ explores the emotional dimension of this activism through the study of transnational symbols like the green scarf. Scholars agree that these strategies coexist, creating synergy among them.^9,13^

### Feminist mobilisation for legal, safe, and free abortion in Ecuador through the tactical-strategies lens

Scholarship on collective action for abortion rights within Ecuadorian feminisms has highlighted the coexistence of two broad strategic orientations, rooted in the Latin American feminist divide between institutional and autonomous feminism. The divergence between institutional and autonomous feminist strategies became particularly visible during the drafting of the 2008 Ecuadorian Constitution. Maldonado^19^ shows that institutional feminists, those operating within state structures or international and non-governmental organisations, relied on conventional lobbying within the Constituent Assembly and aligned themselves with the government’s anti-choice discourse. Meanwhile, younger feminists opted for more provocative actions like the “die-in” – a protest where participants lie down as if dead to dramatise loss and demand action – or breaking into the Assembly sessions bare-breasted to contest the criminalisation of abortion and demand regulatory frameworks ensuring safe access to abortion.^20^ This shows that feminist strategies surrounding abortion and sexual and reproductive health rights have been neither homogeneous nor static, and that within feminist institutional spaces, abortion has not always been on the agenda. For example, during the 2013 debates on the decriminalisation of abortion in the COIP, institutional feminist negotiations with patriarchal power structures led to the exclusion of abortion from the gender agenda.^21^ However, outside institutional spaces, autonomous feminist movements used alternative framings to advocate for free abortion. Feminist activists advanced the notion of life management, arguing that abortion legalisation should be understood as a practice of cultivating life in contexts where capitalism has “rendered” motherhood increasingly precarious.^22^ Although this framing did not result in legal reform, it stimulated public debate, particularly in the media.

Scholars have also highlighted the embodied nature of the repertoires through which abortion has been politicised. Zambrano^23^ analyses the politicisation of abortion through the public exposure of women’s bodies during protests, while Maldonado^24^ underscores how feminist practices of abortion accompaniment enable women to reclaim bodily autonomy and transform sexual politics. More recently, Cevallos and Varea-Viteri^25^ have demonstrated that the Green Tide movement, amplified through social media, has permeated community discourses and facilitated access to abortion information among young Indigenous women, enabling them to exercise this right collectively.

In this context of a divide between institutional and autonomous feminist movements, we use the lens of tactical strategies to draw attention to the fact that abortion brings to the table certain representations of the body that dispute the meanings of life, motherhood,^1^ sovereignty,^24^ and care.^26^ The body acquires a central place in feminist struggles, within which hegemonic meanings defining what the body should be are contested.^27^

In this study, we investigate feminist practices related to abortion and trace their complexity from their politicisation in 2006 until 2023, two years after abortion in all rape cases became decriminalised in Ecuador. We build upon Lugones’s^6^ concept of tactical strategies to identify: (1) the discursive and embodied practices that construct and politicise the aborting body within feminist advocacy for legal, safe, and free abortion in Ecuador; (2) the effects of these practices; and (3) feminist challenges to conservative discourses that, in some cases, appropriate their rhetorical strategies. In the current context of global threats to abortion and sexual and reproductive rights, ensuring that abortion is free, legal, and safe for everyone who needs it is more urgent than ever. Learning from the tactical strategies used by feminist organisations, particularly their strengths, but also their potential limitations and contradictions, can help to make feminist advocacy stronger and more effective in the global struggle for abortion rights.

## Methodology

### The place of the body in research: materials and analysis

This article is part of a larger study on feminist strategies and the body.^27^ We understand bodies as produced by power and also with the potential to contest it. In this paper, we conceptualise the political body following Esteban: “An articulated set of representations, images, ideas, attitudes, techniques, and embodied behaviours, a specific bodily configuration consciously or unconsciously promoted by a social movement, in our case feminism, which takes shape individually and collectively” (p. 65).^28^ Using participant observation and documentary reviews of texts written by feminist organisations in Quito, Ecuador, we analysed how different representations of the political body have emerged from feminist practices and discourses.

The data used in this analysis consists of reports and images from public demonstrations. CC and PG conducted participant observations of public demonstrations organised by feminist collectives between 2017 and 2023 in Quito (the capital of Ecuador), including: (1) demonstrations supporting the decriminalisation of abortion (September 28, 2019 and 2023); (2) demonstrations on International Women’s Day (March 8, 2017–2020); and (3) demonstrations on the International Day for the Elimination of Violence Against Women (November 25, 2017–2020). These are the largest annual feminist demonstrations in the country. In addition to these demonstrations, CC and PG also conducted participant observations during other academic, activist, and artistic events related to abortion and sexual and reproductive rights.

We also analysed items of grey literature on the topic of abortion that were developed between 2006 and 2022 by feminist and other activist organisations and by non-governmental organisations that advocate for abortion rights in Quito. These documents included: (1) four feminist periodicals: *Conmujeres; CPJ SinXtremos; Flor del Guanto,* and Revista Desafiando* – in addition to the special issue on abortion in relation to the campaign to decriminalise abortion in all rape cases, which is found on the website of Fundación Desafío; (2) a zine,^†^ entitled *Comadreando*^‡^ from *Las Comadres* (The Co-mothers); and (3) a blog, entitled *Salud Mujeres* from *Colectiva Salud Mujeres* (Women´s Health Collective). We chose these sources because they are the main outreach materials about abortion produced by Ecuadorian feminist pro-abortion rights organisations and are publicly available. Finally, we analysed reports on abortion that were produced by feminist organisations, as well as public declarations/manifestos that were made as part of public demonstrations for abortion decriminalisation, and statements by feminist organisations in relation to the legal reforms to decriminalise abortion in cases of rape. We approach this material through the concept of tactical strategies, understood as ways of producing meaning and passing on knowledge as forms of resistance. Part of the material was identified by one of the authors during her ethnographic research on Ecuadorian feminist movements and body politics. Building on this initial corpus, the research team subsequently expanded the dataset by searching for additional materials related specifically to abortion, including content from social media platforms and informational resources produced by various feminist abortion collectives in Quito.

The gathered materials included texts, pictures, and videos, and we looked for both the discourses (texts) and practices (observed) that positioned the body at the centre. That is, we enquired into the sedimentation of discourses in bodies, – understood as the layers of language, norms, rules, and cultural meanings that accumulate over time and inscribe themselves in corporeal practices,^29^ with a focus on the processes of embodiment by which the social is inscribed in the body. In doing so, we analysed the body as both “a material process of social interaction” (p. 25),^28^ and a signified experience.^30^ We focused on that which links the bodily with the discursive, approaching the body as both a product and a producer of social practices.^31^

We put this into practice by using Braun and Clarke’s reflexive thematic analysis,^32,33^ taking a discursive approach and following an adapted version of the six steps they describe. We started with (1) an in-depth, repeated reading and exploration of the gathered material, which included texts, observation reports, and images from the demonstrations that had been previously analysed in a former study. This was followed by (2) coding the material into descriptive candidate themes that reflected the different tactical strategies, then (3) developing candidate (initial) themes and writing descriptions of these themes that indicated how each tactical strategy enabled different body representations and brought up different effects, as well as pairing the candidate themes with excerpts from the text and images that exemplified each theme. We then (4) went back to the material to review whether the candidate themes reflected and incorporated the material, and revised as needed. Finally, (5) we continued to discuss and refine the candidate themes until we reached final agreement regarding the structure and assignment of the themes and their labels. In summary, we followed Braun and Clarke’s six steps, with the only difference being that writing formed part of the analysis and was not a separate final step.

Ethical approval was not required since the analysed material was all publicly available. The images presented have been edited to avoid the possibility of identifying participating individuals. The materials we used were copyrighted, with a statement indicating that their use is permitted as long as the source is properly cited.

### Reflexivity

Of the three authors, CC and PG are located in Ecuador, while IG lived in Ecuador from 1994 to 2010, and is now located in Sweden. We all identify as women and feminists, and we all conduct feminist research, but we come from two different disciplinary backgrounds: social psychology (PG and CC) and public health (IG). The idea for this article was generated and the lead was taken by the Ecuadorian researchers on the team (CC and PG), who also collected the data, while IG joined in the data analysis and writing of the paper. The article is part of research that the team is conducting on abortion and feminist movements in Latin America. The fact that all three authors are feminist and pro-abortion rights and that CC and PG are active in the feminist movement in Ecuador has influenced the choice of conceptual standpoints and methodology and has facilitated the gathering of material.

## Results and discussion

### Reparation, rights, and dissidence

From analysis of the material, we developed three themes that reflected major tactical strategies used by feminist movements in their struggle for legal, safe, and free abortion in Ecuador: reparation, rights, and dissidence. Each of these tactical strategies enables different representations of bodies and has different effects. These tactical strategies are neither representative of a chronological progression nor the monopoly of one organisation; instead, they overlap and are used at different times and places, sometimes jointly and sometimes independently. They also need to be understood in relation to the changing political and contextual circumstances. We do not claim that some of the tactical strategies are better than others, but only that each leads to different effects and that these effects open up certain possibilities while closing others.

### Abortion as reparation: the body of the child–mother victim/survivor of rape

The tactical strategy of reparation represents abortion as a way of (partially) repairing the harm done to a victim, and goes hand in hand with the representation of the body of the victim/survivor.

In Ecuador, the body of the girl-child victim of rape/incest has been at the forefront of most of the public campaigns advocating legal changes to decriminalise abortion in cases of rape. This victim returns us to the concept of the “ideal victim”^34^ in the sense that she cannot be held responsible for her pregnancy: the pregnancy is the result of a sexual act that took place against her will. In this case, youth is a marker of vulnerability and reinforces the victim’s innocence; teenage pregnancy and sexual violence are brought together and presented as compelling arguments for extending the exceptions for legal abortion to cases of rape to offer reparation for victims. The evidence upon which these arguments are based includes the heart-breaking testimonies of the victims. For example, in the magazine *Conmujeres,* Leyla Escobar writes:

“*They were timid moans, almost imperceptible, but they were moans that woke me up […]. In the other bed, in the hospital ward, there was a girl of about 12 years old crying bitterly […]. Her story was heart-breaking. Her employer had raped her and she has become a mother at the age of 12*.” (p. 13)^35^

These testimonies are further backed up by studies providing evidence that sexual violence against girls is common in Ecuador and has harmful effects. For example, the study “Stolen Lives,” published by *Fundación Desafío* in 2016, focused on 15 girls who were “raped and pregnant before the age of 14” to explore the harmful health consequences of mothering after rape.^36^ Statistics on teenage pregnancies and symbols of childish, gendered bodies are brought together in feminist demonstrations to reclaim abortion as a form of reparation for these ideal victims. Figure 1, seen at a demonstration on March 8, 2020, shows the figure of a girl child with the text: “Eight girls younger than 14 are forced to *give birth* [emphasis added in the original] every day in Ecuador”. This picture connects with the slogan heard on the 28th of September marches: “Girls are girls, girls are not mothers”.

**Figure 1.**
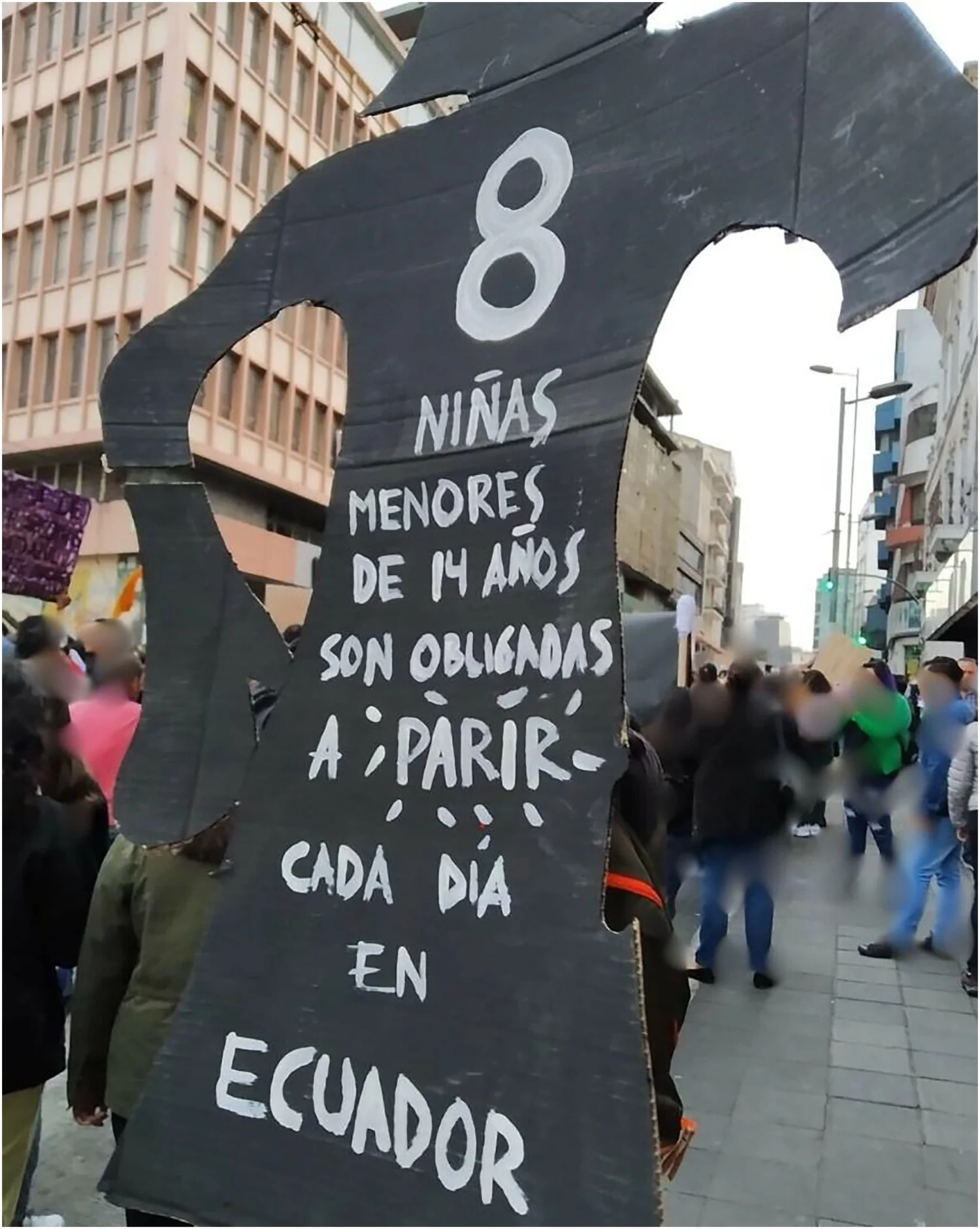
Poster at the demonstration 8th March 2020. Source: Authors’ file

Within this context, we would like to focus on the campaign of the “*guaguas de pan*” (bread children), which went hand in hand with the *hashtags* #laverdaderaguagua ecuatoriana and #EsConstitucionalDecidirSobreNuestroCuerpo. The “*guagua of bread*” (“*guagua*” is the word for child in the Indigenous language of Kichwa) is a traditional food prepared for the Day of the Dead, and consists of the figure of a small child made of bread. Figure 2, below, shows how feminist advocacy re-interprets the traditional “*guagua de pan*”, turning it into a pregnant “*guagua*”. The text of the campaign, launched as part of a package of advocacy strategies to generate pressure to decriminalise abortion in cases of rape, reads: “The Ecuadorian court should be brave, as regional courts have been, and address the root issue, which is abortion in cases of rape”.^37^ Feminists from Fundación Desafío delivered the pregnant bread children to the nine judges of the Constitutional Court to encourage them to rule in favour of abortion in cases of rape.^38^ (October 29) In the pregnant “*guagua de pan*,” two situations that it seems impossible to find coexisting in one body are brought together: the paradox of childhood and motherhood within the same body. The campaign was aimed at visualising the hidden burden of sexual violence in the life trajectories of girls and women and to shed light on the culture of rape that leaves incest unpunished. Motherhood embodied in the infantile body of a girl provokes the audience; in a context in which motherhood is, in general, sanctioned and idealised as a woman’s destiny,^39,40^ in the campaign becomes reconfigured as a form of torture when it is embodied in the girl child.

**Figure 2.**
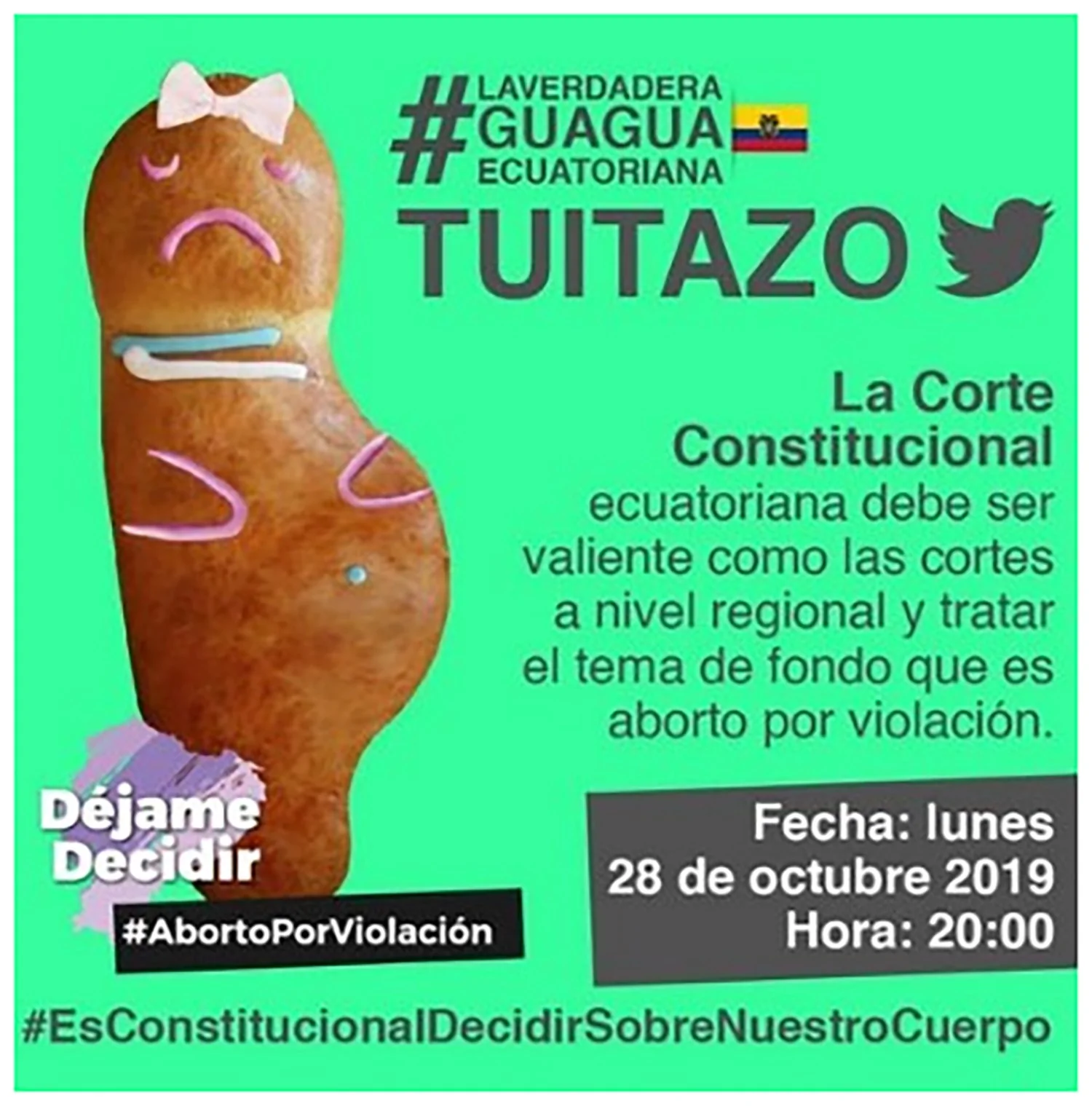
Image of the tweet #It's constitutional to decide about our body. Source: Abortion by Rape Campaign. Fundación Desafío. https://bit.ly/3HkN1nO

As the text of the poster argues, this representation of the (innocent) sad victim requires a “brave” response from the court to repair the abuse committed. The way to repair this abuse is to address the issue at its core: to make it legal to have an abortion in cases of rape. This body representation of the “ideal/innocent victim” also offers a strong counterargument to the flagship argument of the anti-abortion forces that the fetus is an innocent victim with no decision-making power and is in need of protection. The fetus is no longer the only innocent victim here; the girl child who is forced to become a mother is also a victim. Moreover, while the fetus is a potential victim, the girl-child victim of rape who is pregnant is a victim here and now, with no decision-making power and in need of protection. The tactical strategy of reparation represents abortion as a way of protecting innocent victims. Its strength lies in reconfiguring an anti-abortion argument (the protection of the innocent unborn) into a pro-abortion one (the protection of the innocent, pregnant, girl-child).

The tactical strategy of reparation has led to progress in other spheres, such as protecting women from gendered violence. When it comes to abortion, appealing to the representation of the girl victim of sexual violence gained traction with the regional campaign *Girls not Mothers*. Planned Parenthood Global, Amnesty International, the *Grupo de Información en Reproducción Elegida* (GIRE – Information Group on Reproductive Choice), and the *Consorcio Latinoamericano contra Aborto Inseguro* (Latin American Consortium against Unsafe Abortion) have been working together on this campaign since 2016. The campaign had repercussions in the traditional media, showing that despite acknowledging the abuse of minors, legal frameworks continue to mandate involuntary motherhood, criminalising those who seek to terminate forced pregnancies.^41^

As always, this pro-abortion campaign was mobilised side by side with the anti-abortion religious fundamentalist campaign against the so-called “gender ideology” entitled “*Con mis hijos no te metas*” (“Don’t touch my kids”). This campaign and the demonstrations it arranged appear to have been the most massive act of anti-rights groups in Ecuador in recent years, by articulating opposition to comprehensive sexuality education, LGBTIQ + rights, and feminist movements with an idealised defence of the traditional family and national moral values. However, it was built upon a sustained history of conservative lobbying, political agreements at the national governmental level, economic power, alliances between hegemonic churches, and support from conservative social sectors.^42^ The tactical strategy of reparation operates as a counter-conservative discourse, deploying an ironic gesture that draws upon familiar symbols – such as the figure of the innocent child – and disrupts common sense by exposing pregnancy in a child’s body. Ecuadorian feminist coalitions mobilised the representation of the victim to appeal to society during the time of the Constitutional Assembly in 1998 and then again in 2008,^43,44^ particularly for women as mothers and victims of gender-based violence. This tactic re-emerges at a later stage and articulates a linkage between the fight against sexual abuse in schools and claims to abortion rights in cases of rape. Emphasising that girls are among the victims of sexual violence was central to generating social consensus regarding the magnitude of the problem.

An illustrative example of this link occurred in July 2018, when a collective of survivors of sexual violence launched the campaign “*Seremos las últimas*” (“We Will Be the Last”) to publicly expose the sexual abuse perpetrated by an Olympic gymnastics coach at a well-known sports centre in northern Quito.^45^ As a result of this campaign, the facility was shut down, and a formal investigation was initiated to secure redress for the victims. The demand for guarantees that there would be no repetition of such abuse emerged as a central slogan in the pursuit of justice, particularly in contexts where perpetrators have historically enjoyed impunity.^27^

The position of the victim is foregrounding a position that appeals, above all else, to the future. This is evident in the emblematic case of Norma, which was brought before the United Nations Human Rights Committee on May 29, 2019. Norma was a 13-year-old girl who was forced to continue with her pregnancy (a consequence of incest) despite having requested an abortion within the public healthcare system due to the risks the pregnancy posed to her health and life (an exception under which abortion was legal in Ecuador at the time of the request).^46^ The power of this case lay in the fact that Norma was an “ideal” victim. Her case led to the UN Human Rights Committee’s resolution, published on January 20, 2025, that condemned the Ecuadorian state for violating Norma’s right to life and acknowledged that she was a victim of torture and discrimination. The case was brought with the aim of preventing this from happening again and to force changes that would improve the lives of future Ecuadorian girls. Therefore, the right to have an abortion is a right to protect the future life of the victim or of other potential victims. Only then will the suffering of innocent victims have a purpose and become the seed of progress; it becomes feminist solidarity. The reparations in Norma’s case consisted of financial compensation to support both her education and that of her child, as well as public acknowledgement of the responsibility of the state.

Thus, the tactical strategy of reparation relocates the responsibility for the violence suffered by these victims from the individual aggressor to the complicit state. The state is responsible for allowing sexual violence against girls and adolescents to exist and persist; it is responsible for its failure to protect children and for not offering reparation to the victims since the possibility of resorting to legal, free, and safe abortion is not guaranteed.

### Abortion as a legal right: the body of a woman citizen

The second tactical strategy is that of advocacy for abortion as a right. In this case, the political body representation being mobilised is of the woman citizen who is a responsible adult, with the capacity to exercise sexual and reproductive rights, and who needs to be able to count on the possibility of having an abortion if her health or life (project) is in danger. This tactical strategy is part of a logic of human rights that advocates “for our right to decide” (Figure 3), as shown on the banner depicted below, which was used in one of the demonstrations for abortion decriminalisation.

**Figure 3.**
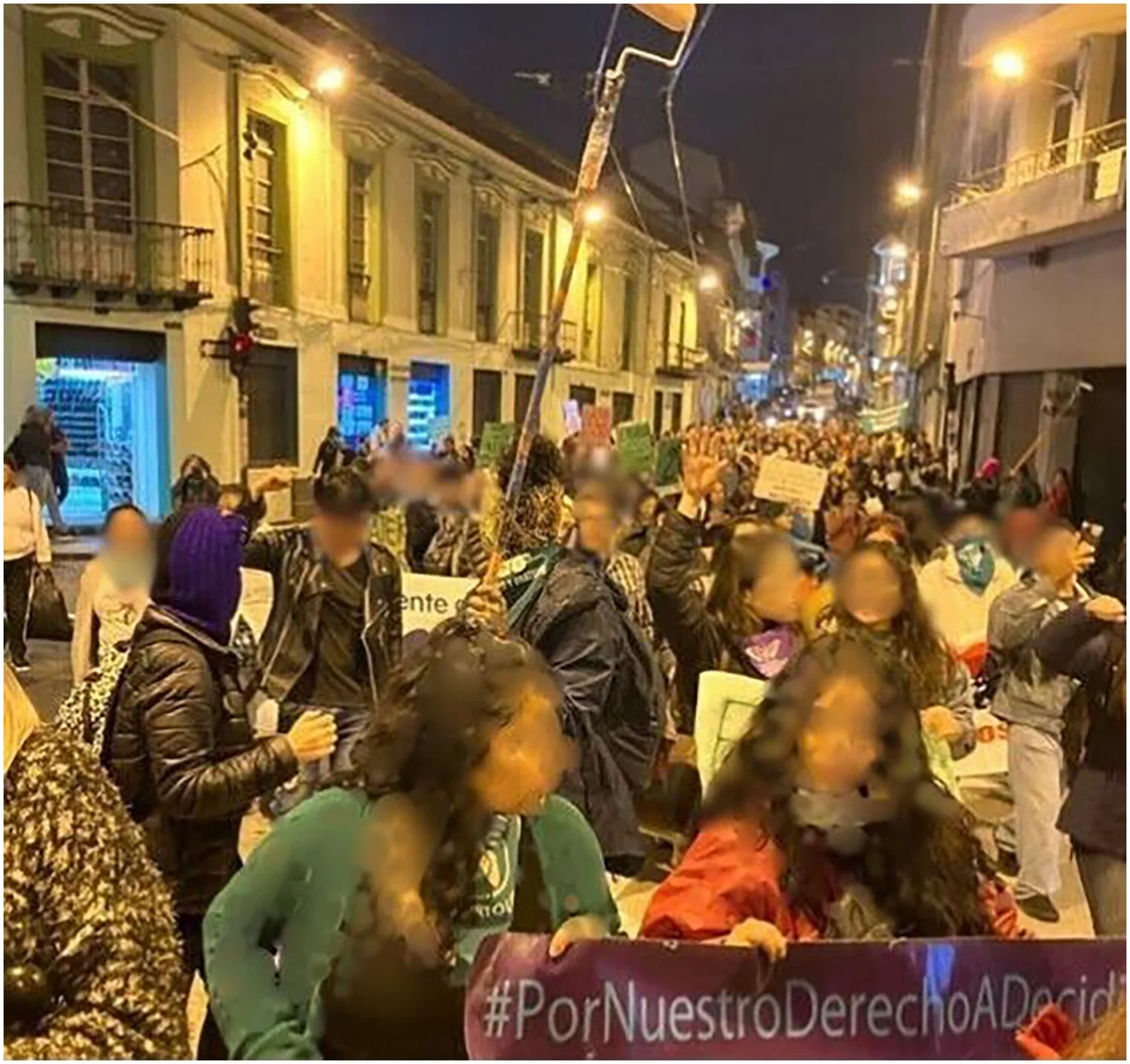
March for the decriminalisation of abortion, September 28, 2023. Source: Authors’ file

Prior to explicitly incorporating abortion into feminist demands, this tactical strategy was situated within the broader defence of sexual and reproductive rights. During the late 1990s, both the broader social movement and the women’s movement played an active role in constitutional reform processes and in the revision of the Ministry of Health’s reproductive health regulations. As a result, emergency contraception was formally recognised as a standard contraceptive method. In parallel, in 2002, the Public Prosecutor’s Office incorporated a gender-sensitive approach into protocols for the care of survivors of sexual violence. This shift entailed a mandatory duty to provide women who had experienced rape with clear information about their reproductive options, including access to emergency contraception.^47^

However, this progress was subsequently contested by conservative Catholic groups. The opposition became particularly visible during public debates surrounding the commercialisation of the emergency contraceptive pill Postinor (the “day-after pill”), which set pharmaceutical companies against conservative actors. Although these groups succeeded in blocking the commercialisation of one specific brand of emergency contraception, other brands continued to be available on the market.^22^

In this context, the periodicals *Desafiando* (Challenging) (2002–2008) and *Conmujeres* (With Women) (2006) promoted the use of emergency contraceptives and the establishment of protocols regulating their use in cases of rape, framing these measures as essential both to protect women from unwanted pregnancies and to strengthen women’s empowerment. Within this framework, access to abortion is articulated as an integral and indispensable component of a broader continuum of sexual and reproductive rights. This understanding is encapsulated in the *Conmujeres* (With Women) slogan, “sexual education to decide, contraceptives to prevent abortion, legal abortion to prevent death”,^48^ which emerged in Argentina during the National Campaign for the Right to Legal, Safe, and Free Abortion.^49^ This selective framing reflected strategic considerations of social acceptability and political feasibility.

To legitimise this tactical strategy grounded in rights, abortion is articulated as an integral component of a broader framework of sexual and reproductive health and rights, underpinned by the claim that unsafe abortion constitutes a public health problem. Within this framing, abortion as a means of preventing maternal mortality emerges as a central argument, powerfully captured in the slogan “I was not born a woman to die for being a woman” (Figure 4).

**Figure 4.**
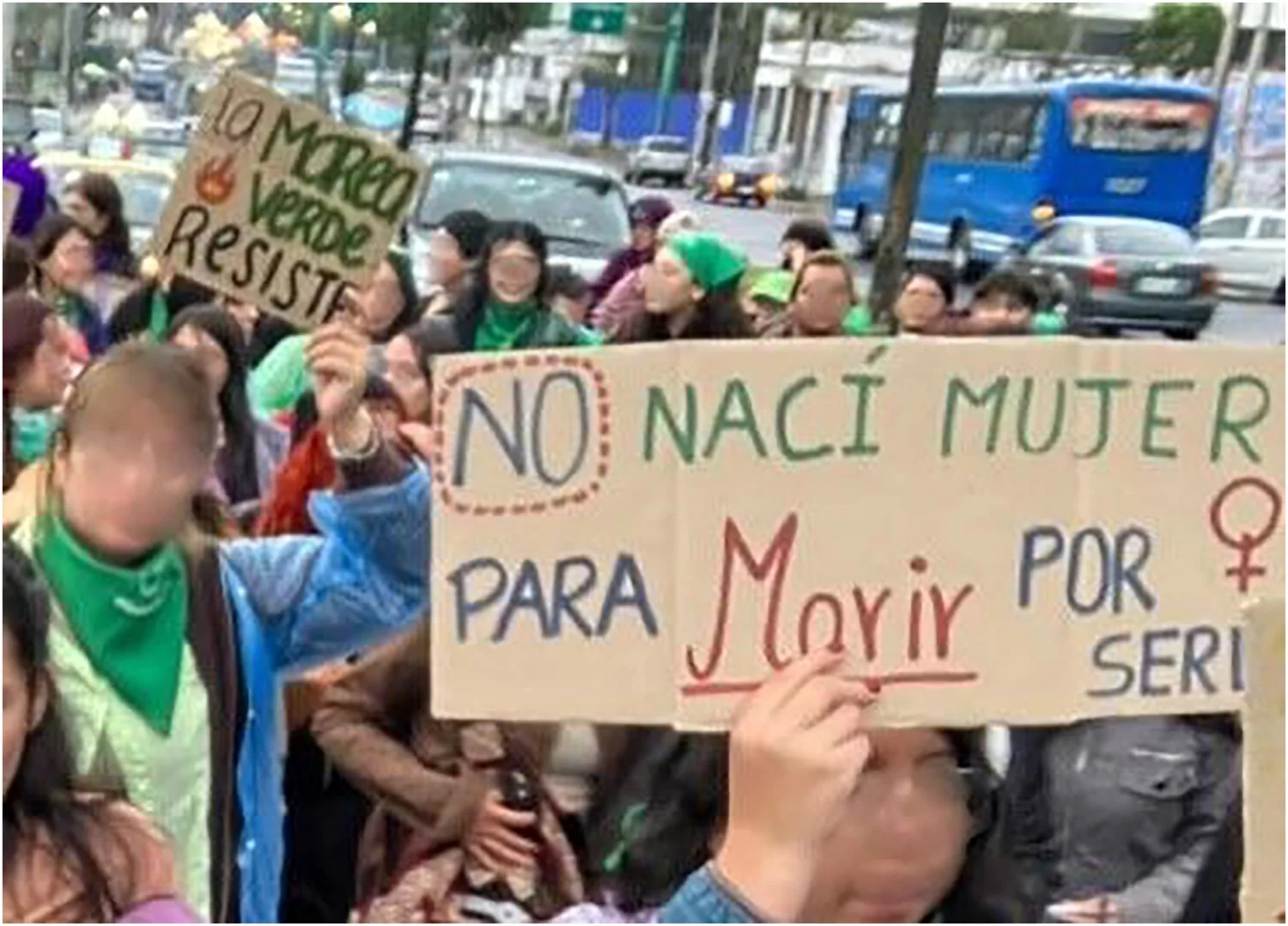
“I was not born a woman to die for being one”. March for the decriminalisation of abortion September 28, 2023. Source: Authors’ file

Through the tactical strategy of claiming reproductive rights and the right to abortion exception as framed in legally binding national policies, feminists defend therapeutic abortion. As explained in the periodical *Desafiando*, “Therapeutic abortion is an obstetric emergency, therefore, preventing women’s access to abortion violates the right to health and what is enshrined in the Constitution” (p. 6).^50^ This tactical strategy draws upon biomedical discourses and operates within the legal possibilities presented by therapeutic abortion when a woman’s health is at risk.

Until 2015, the health exception (*causal salud*) lacked specific health regulations or clinical practice guidelines to operationalise this right and protect health professionals who performed legal terminations of pregnancies.^51^ It was through the advocacy of feminist movements that the Guidelines for Therapeutic Abortion were developed. Within this framework, it became possible to initiate processes of sensitisation and training within both the public and private healthcare systems.

By linking abortion to rights-based claims, this approach frames the procedure in terms of morality, justice, and dignity.^52^ In doing so, it responds to conservative objections centred on the protection of life, the imperative of saving the mother’s life, and the legitimacy of conscientious objection by medical personnel.

The tactical strategy of rights is closely intertwined with the strategy of reparation, due to rights claims in Ecuador having become intelligible through the framing of women as victims of gendered violence requiring state and healthcare intervention. This articulation was central to the decriminalisation of abortion in cases of rape, where the figure of the woman citizen was mobilised alongside that of the victim of sexual violence. While this framing enabled significant legal advances, it also reinforced a victim-centred approach that, rather than transforming social relations, has tended to reproduce the patriarchal order and traditional gender roles.^53,54^

The body of the woman citizen also encompasses a paradoxical effect: it opens the possibilities for abortion by contextualising it within a broader continuum of sexual and reproductive rights and by (slightly) expanding the number of exceptions within which abortion is no longer illegal. But it also limits the opportunity to question abortion exceptionalism^55^ – understood as the construction of abortion as a morally and medically distinct practice subject to differential regulation – because it relies on the medical and legal system to regulate women’s lives, sexuality, and reproduction. This tactical strategy also places a lot of hope in state institutions to authorise access to abortion when we know that, even in contexts with progressive laws, the existence of such laws does not guarantee access and, even less, equal access.

The key limitation of this tactical strategy is that it mitigates the discomforts but does not uproot the processes of domination that perpetuate abortion exceptionalism.^56^ Likewise, the body representation of the woman citizen prescribes who is entitled to these rights – the woman/mother, heterosexual or victim of gender violence, who is urban, with access to health services, educated, and a responsible user of contraceptives^27^ – and who may be excluded. As proponents of reproductive justice would argue, rights are never exerted in a vacuum.^57,58^ While such a discourse could open up the possibility of a sense of entitlement to public goods and their legal protection, motivated by a particular view of women as full and equal citizens, it also reinforces the power of the medico-legal system. In summary, the discourse constructs a subject who is continually performed in interactions with public services: women appear as citizens, yet their citizenship is restricted and conditional, defining the very conditions of ability and inability to exercise rights.

### Abortion as dissidence: sovereign bodies

The third feminist tactical strategy uses abortion as a flagship for struggle and rebellion. Opposing victimisation and the attempt to expand the regulation of abortion within the limits of the institutional system, this tactical strategy means breaking away from the system while demanding the total decriminalisation of abortion, beyond exceptions.

What distinguishes this tactical strategy from the two previous ones is its rejection of the state as a guarantor of reparations and rights. From this tactical strategy emerges the sovereign body, which appropriates reproductive technologies to access abortion without the need to resort to legal approvals or follow the protocols stipulated by law. The sovereign body is also a feminist body, irreverent, a naked torso, a political body that exercises sovereignty beyond the tutelage of medical, legal, and religious powers. Elsewhere, CC has proposed that this body can also be called the body-territory,^27^ a tactical strategy that articulates a sexual and reproductive rights discourse and the political struggles of Indigenous women against racist, extractivist^§^ and patriarchal violence.

The tactical strategy of dissidence aims not only at legal decriminalisation but also, and especially, at the social decriminalisation of abortion. This is proposed through transforming the imagery surrounding abortion, redefining it as an act of feminist rebellion; for example, it is written in the zine *Comadreando* No. 2 that “I can be one of the first in my family who will not have children by imposition but by choice […] It is an act of self-love” (p. 12).^59^

Within the frame of grassroots feminist accompaniment of self-managed abortion,^17^ abortion is reconfigured as an act of shared learning and of commitment to one’s own life project. These networks – for example, the network Comadres that offers information about how to accomplish a self-managed abortion and accompaniment during and after the procedure, and is also the author of the fanzine quoted here – are at the forefront of destigmatising abortion through providing information, accompaniment, and politicisation of self-managed abortion. Within this tactical strategy, drugs, such as misoprostol and mifepristone, are redefined as a form of empowerment that is used independently of medical guardianship, as the slogan chanted in the demonstrations proposes: “And if the State threatens us, we will be happy, we will, we will be happy, aborting at home”.

In Ecuador, accessing misoprostol without a medical prescription is not possible; therefore, since 2008, information about how to acquire the drug has been provided by the telephone line initiated by the *Colectiva Salud Mujeres* and, later, through the direct distribution of pills and by feminist accompaniment of abortion provided through the feminist collective *Las Comadres*. As the poster in Figure 5 proclaims, this tactical strategy does not rely on the legal and/or medical system for accessing abortion, an approach that, according to Rosenberg,^60^ constituted a breaking up with the heteronormative patriarchal order of the legal system.

**Figure 5.**
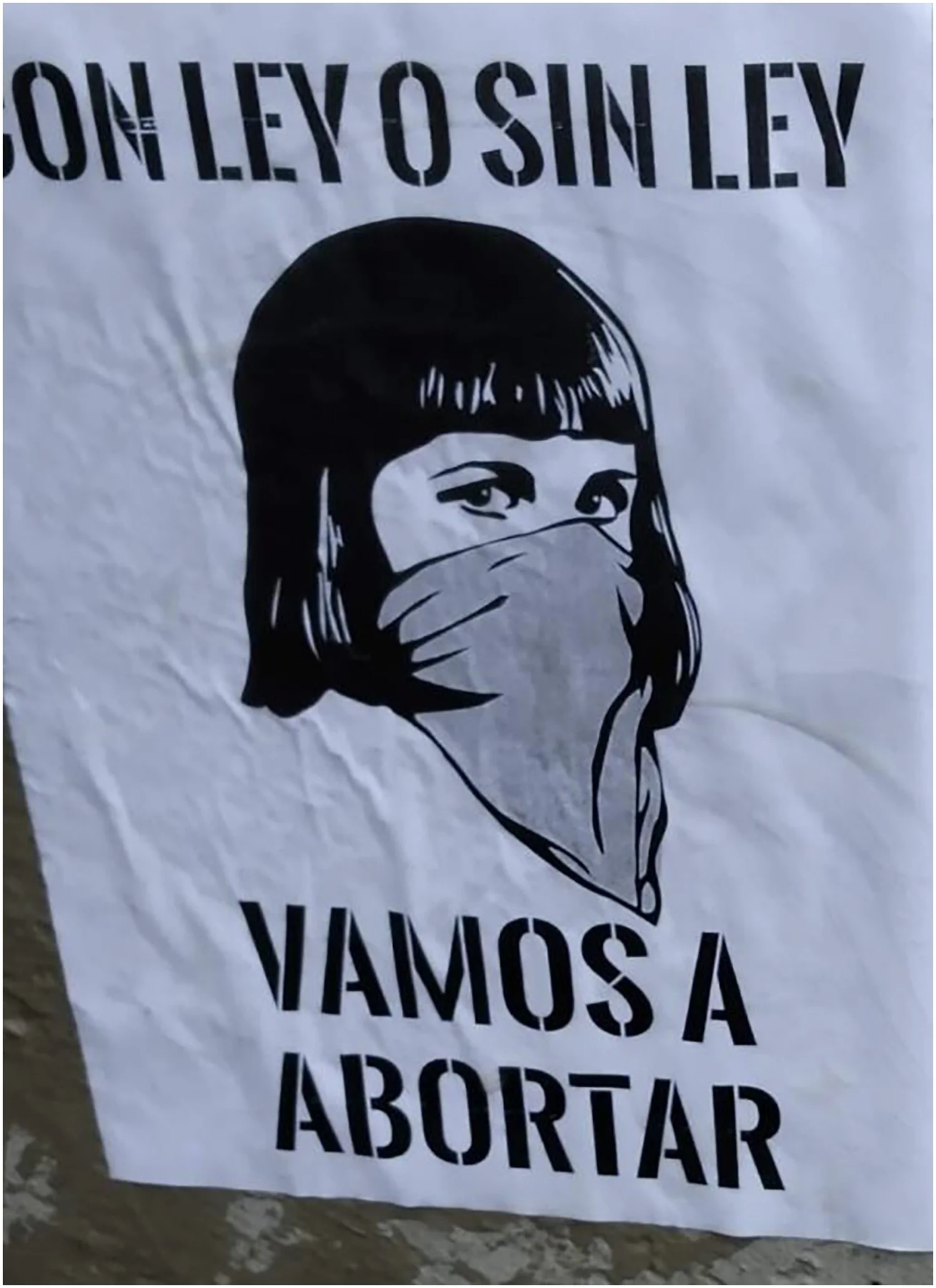
With law or without law we will abort. March for the decriminalisation of abortion, September 28, 2019. Source: Authors’ file

The green handkerchief that has become a symbol of the feminist fight for free abortion in Latin America is used in the poster as a mask, not primarily to hide the identity of the protester but as protection from the police gassing and repression that is expected during demonstrations. Dissident feminist activists are not calmly advocating for incremental legal changes through negotiations and dialogue with politicians and those in power; they are protesting in the streets against the established order that constrains their sovereignty over their own bodies.

The sovereign body is expressed in public space with bare breasts. Nudity exposes the bodies that abort, presents the bodies that challenge the sexual order, and is, therefore, part of the repertoire of feminist action (see Figure 6). These feminist bodies have been present in both the debates on decriminalisation in the National Assembly and feminist marches and performances for the legalisation of abortion such as the 28 September demonstration. These bodies confront the conservative interpellation of mandatory motherhood and reproductive sexuality with proclamations of joy and freedom.

**Figure 6.**
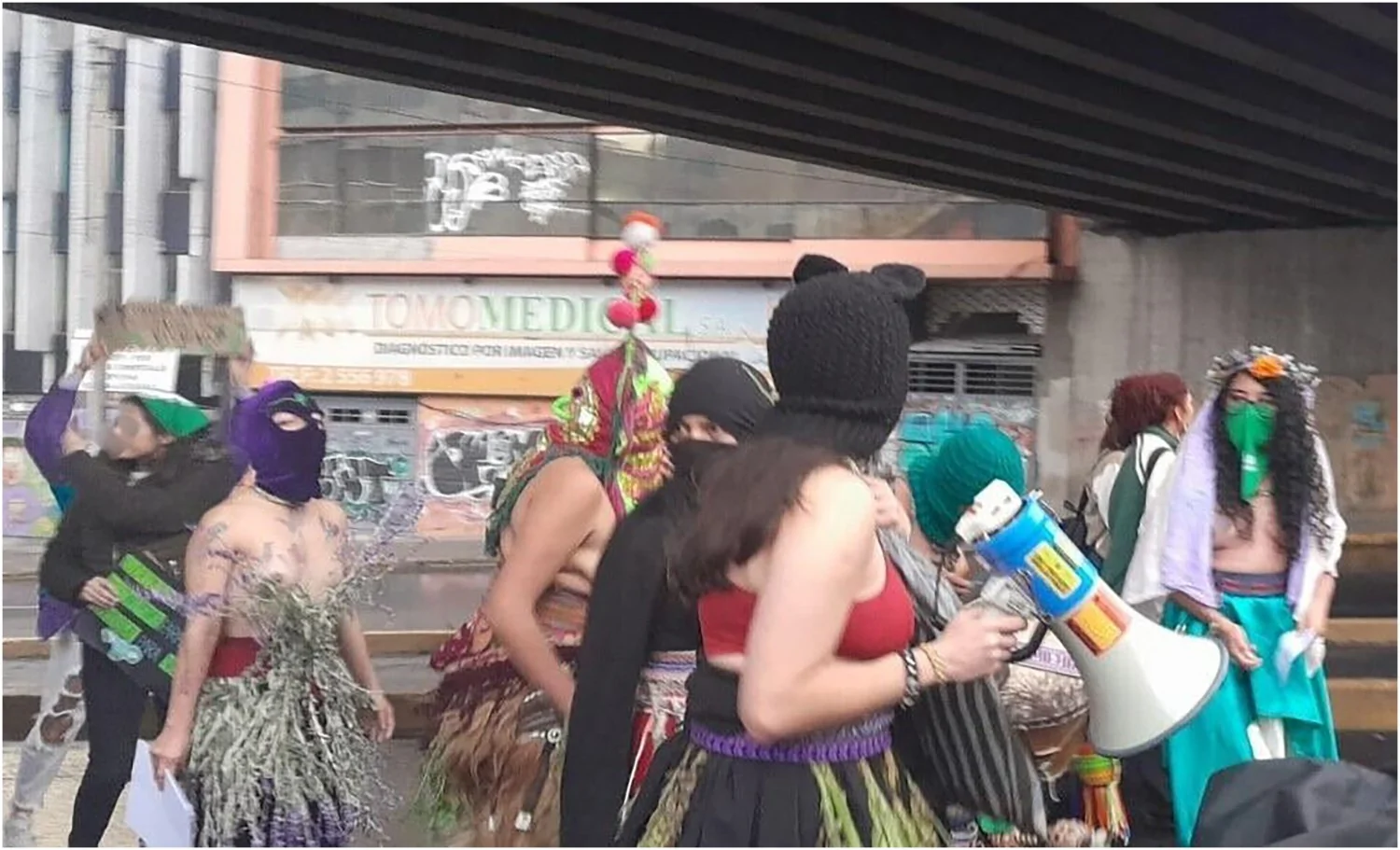
Bare breasted and hooded women with bunches of lavender and rue. March for the decriminalisation of abortion, September 28, 2023. Source: Authors’ file

The sovereign body is trans-inclusive in the sense that it also embodies abortion in bodies that exist beyond the category “woman,” with the inclusion of terms such as “pregnant person” and “person with the capacity of becoming pregnant”. Beyond the rights approaches, this tactical strategy takes an intersectional approach to recognise that not everyone can exercise their rights under the same conditions. The sovereign body is multiple in its presentations, including women who are imprisoned for having abortions, young activists within networks of feminist accompaniment for abortion, young women protesting on the streets, Indigenous women, and sex-gender dissidences.

This tactical strategy does not seek incremental legal changes that may give pregnant people a little more room to manoeuvre but rather seeks to ensure women’s and pregnant people’s capacity for self-determination. This requires breaking up with the current system, as explained in the blog of the Colectiva Salud Mujeres (n.p.)^61^:

“We do it because we believe in the capacity of each woman to self-determination, because we understand the importance of counter-information to destroy the system that oppresses us, because we know that only solidarity among women can change our lives here and now.”

The sharing of information (i.e. about self-managed abortion) aims to reduce inequities (based on class, social capital, racialisation) through the democratisation of knowledge about the body and, consequently, the power to decide. Beyond understanding sexuality and reproduction from a rights-based perspective, this tactical strategy is more in line with a reproductive justice approach.^57,58^ Reproductive justice highlights that not all women and girls have the same opportunities to make reproductive choices, and that not all women are directed towards the same choices. First, it questions the homogenisation of women and their reproductive “choices” that disregards the impact of racism, class, and other forms of oppression on women’s opportunities to exercise and enjoy their rights. Second, it problematises the dominant liberal discourse about “individual rights and the marketplace of choices,” arguing that rights and choices are frequently denied to marginalised members of society.^57^ This strategic tactic involves feminists’ instrumental use of the state as a site for expanding rights within neoliberal contexts of institutional weakness. By re-centring the collective, feminist actors mobilise community networks to contest dominant meanings, secure the legal right to provide information, and channel dissent through institutional interstices, thereby translating collective strength into concrete change.

As Ramón and Cavallo^62^ found, access to information represents a challenge to the monopoly of biomedical and pharmaceutical devices. Network collectives that provide feminist accompaniment for abortion build on solidarity and shared knowledge, and they construct abortion as self-management, without the need for hospitalisation or medical control.^17,63^

Within this tactical strategy, abortion is not a goal in itself but rather a means to reclaim the right to sexual pleasure without fear of the consequences. As Nancy Carrión stated in *Flor del Guanto* magazine, “We were questioned in the public debate: ‘Do you have sex only for pleasure?’ And we answered yes, because pleasure reminds us that we are sensitive bodies, pleasure reminds us to love ourselves and to relate healthily and honestly with the world” (p. 41).^64^ The dissident body claims pleasure is good and important and a way to reclaim power.

Another crucial element of this tactical strategy is recognising plants as allies that link ancestral and current abortion practices. Plants connect abortion struggles and Indigenous wisdom; they open the possibility to talk about abortion from the perspective of Indigenous and Black people. “Abortion is ancestral; guilt is colonial” sing feminists, hooded and adorned with bunches of rue and borage during demonstrations, two plants that have been used as abortifacients for generations (Field diary, March on September 28, 2023; see Figure 6).

Ancestral practices of body sovereignty are being recovered to decolonise the practice of abortion and reconfigure it as connected to the historical resistance of Indigenous nations. As shown in the artwork “Bitter Infusion” by the *Cholas Valientes* (Brave Cholas^¶^) collective and exhibited as part of the collective exhibition “Plants of Power” at the Metropolitan Cultural Center:

*RUDA in small doses is a good companion.*
*Your closed hand is the perfect measure,*
*in a cup when the water boils,*
*for a maximum of seven days, if menstruation does not come*.^65^

This tactical strategy questions the control exerted on bodies by religious powers, which for centuries have used guilt as a disciplining mechanism to control women’s bodies. The sovereign body denounces the fake secularity of the state to unveil patriarchal complicities in the control of female sexuality. In the 2023 Manifesto for the Day of the Legalisation of Abortion, the feminist collectives argued against “the use of guilt as one of the instruments of domination and manipulation of society and religions over our bodies and decisions”.^66^ While the argument of the secular state was mobilised by Ecuadorian feminist movements in the 1990s to defend sexual and reproductive rights and support secular and comprehensive sex education, the tactical strategy of dissidence reaches beyond this by denouncing the intimate subjection through guilt that religion promotes. By re-centring the collective, feminist actors mobilise community networks to contest dominant meanings, secure the legal right to provide information, and channel dissent through institutional interstices, thereby translating collective strength into concrete change.

## Concluding discussion

In this article, we have analysed the various tactical strategies that feminist activists in Ecuador have used to advocate for legal, safe, and free abortion and the political body representations that they create: the tactical strategy of abortion as reparation, which requires the victim/survivor body; the tactical strategy of decriminalisation, which legitimises the citizen body; and the tactical strategy of dissidence that goes hand in hand with the sovereign body. We have not presented them in any hierarchical order but instead have analysed how each of them enables certain possibilities. They need to be understood within the broader political context and the evolution of anti-choice forces. Thinking with Lugones,^6^ these tactical strategies are open-ended and lie between different worlds of sense (the legal, the medical, the cultural) without evident closure or completion.

The three tactical strategies need to be contextualised in the sense that different and changing contexts lead feminist activists to choose and try different tactical strategies. For example, the mobilisation of the girl-child victim body responds to an environment that is sensitised, moved, and acts in relation to children (and fetuses) as vulnerable^67^; the woman-citizen body responds to an environment charged with a discourse of human rights and answers the call of conscious objection; while the sovereign body emerges as a challenge to the passivity and complicity imposed upon the citizen body by the medical and legal apparatuses to guarantee a life of dignity. While different tactical strategies can coexist and complement each other, frictions and contradictions can also emerge when various tactical strategies are employed simultaneously.

The tactical strategy of reparation can be understood as a facilitator, amalgamating diverse organisations around a common minimum; abortions in cases of rape are the lowest common denominator, i.e. the exception that gains support from the widest range of actors. The strategy then broadens the debate and the visibility of the realities of violence against women and girls in the country. The risk in employing this tactical strategy in a context of regression of rights already won is that it creates a hierarchy of victimhood and that the girl-child and the respectable citizen are seen as the legitimate beneficiaries of abortion, to the detriment of those who cannot occupy such subject positions.^67^ Meeting the requirements of the “ideal victim”^34^ can be enormously limiting and may have especially harmful effects for those who do not perfectly fulfil the requirements. We question whether it would be possible to mobilise the tactical strategy of reparation for a victim who is not broken, a victim who is not ashamed of her sexuality, a victim who is not sufficiently “innocent”? If abortion as reparation is only acceptable for those who have been victims of sexual violence, how can we advocate for abortion when those requesting one are not victims (according to the existing legal and social frames) or do not want to consider themselves as victims?

The tactical strategy of legal rights proposes a body that becomes a subject if she can exercise rights. However, this body is contingent upon medical and legal discourse. It is a body that is shaped by institutional frameworks of power, through medicalisation as a by-product of decriminalisation, which keeps abortion procedures under strict medical supervision.^52^ Other forms of governance require that the citizen body appeals for rights and operates within the limits of legal frames; it builds trust in the capacity of the system to expand and secure citizens’ rights. This trust in the system that is established to guarantee rights forgets that not all people are guaranteed the same access or the same protection by the system. The right to abortion as a procedure that ceases to be illegal and is regulated by health policies does not ensure that people who do not belong to the hegemonic classes, ages, sexualities, genders, racialisation, and functionalities will also have that right guaranteed.^57,58^ In a state that discriminates against and violates the rights of people who do not belong to the elites, why should the subordinates expect that access to abortion is an exception to their experiences of discrimination and injustice?

The sovereign body represents abortion as a quotidian and de-medicalised practice that, in a context of compulsory motherhood, requires a network of feminist accompaniment for its realisation.^17^ This is how it sustains a new type of identification: the woman who aborts^60^ to escape the stigma, and who, by aborting, contributes to destigmatising abortion. The sovereign body is also a collective body of blurred boundaries in its connections with plants, women’s communities, and self-managed abortion through pharmaceutical technologies. Its most visible actions have been public protest and grassroots “accompaniment networks” for abortion, which give meaning to the collective actions for the social decriminalisation of abortion and contribute to narrowing the gap between those who require an abortion and those who can effectively access it. Its strength is the recognition of structural inequalities that cannot be resolved through legal and normative changes.^17,63^

The tactical strategy of dissidence and the sovereign body allow for the articulation of feminist activism with anti-racist and sexual dissidence activisms by promoting a decolonising vision of the body that reaches beyond the biomedical paradigm. Feminist accompaniment of abortion seeks not only to enhance access to abortion but also to reconfigure it as a solidarity practice, a good experience without guilt, fear, violence, or control (see also the magazine *Comadreando* No. 2) and that continues outside Ecuador^17,63^; abortion becomes an experience that extends beyond the biomedical. Within the Argentinian context, the effectiveness of accompaniment networks (such as the Socorristas) lies not only in providing technical access to misoprostol but also in fostering an affective shift: moving from the “denunciation of suffering” towards the “affirmation of sovereignty and joy”.^14^ Future research could explore whether the strategy of dissidence in Ecuador is achieving a similar affective transition towards the social destigmatisation of abortion, reaching beyond the courtrooms and into the fabric of everyday life.

Against the mistrust in institutions, the tactical strategy of dissidence builds upon feminist solidarity and rebellion. However, we could also question the reach of such a tactical strategy: how many women can be reached by initiatives that have to be economically self-sustained or that depend upon (decreasing) external funding and drugs commercialised by international pharmaceutical companies (especially in the current context of aid cuts)? Who may be excluded or subordinated by an approach that represents abortion as an empowering and collective process?

In Ecuador, feminist movements have prioritised widening the exceptions for legal abortion to include all cases of rape due to the opening offered by the legislative reform of the Comprehensive Criminal Code and the associated lawsuits that were presented before the Constitutional Court. They succeeded in widening the exception to include all cases of rape (not only when the woman had a disability, as was the case before 2021). On the one hand, we can understand this success as a wedge that could allow expansion to more exceptions in the near future. On the other hand, this success could also become a firewall that makes it difficult to broaden the scope of the law to include exceptions that do not fit within the tactical strategy of reparation for victims. A strategy that attempts to reform the rules of the game through incremental changes in the legal and medical system seems more feasible than a radical shift towards total decriminalisation and de-medicalisation. However, is this progressiveness possible in the current political context, or is it simply an empty promise to convince feminist movements to lower their aspirations?

We recognise that tactical strategies like the three depicted here are also found in other contexts beyond Ecuador. By understanding their effects, we can be aware of the possibilities that each of them creates, as well as the potential drawbacks. Since no tactical strategy is perfect and deploying each one also depends upon the possibilities that each context provides, visualising the potential drawbacks that come with each tactical strategy allows us to plan for ways to minimise these. The different tactical strategies offer the possibility of collectively mobilising in the face of the anti-choice hegemony.^68^ Their potential lies in their plurality and their chameleon-like capacity for transformation and articulation.

The tactical strategies of the Ecuadorian feminist movements for legal, safe, and free abortion open and close diverse possibilities; at times, they articulate and complement each other, but at other times they may contradict each other. Despite frictions (which are necessary in any democratic and pluralist movement such as feminism should be), feminists in Ecuador have managed to keep alive a struggle that does not end when it achieves small gains and persists thanks to the articulation of diverse tactical strategies.

## Footnotes

* *Conmujeres* (With Women) from *CONAMU (Consejo Nacional de Mujeres –* National Council of Women); *CPJ SinXtremos* (Youth Political Coordination Without Extremes) from *Coordinadora Política de Jóvenes* (Youth Political Coordination); *Flor del Guanto* (Angel's Trumpet-flower) from *Casa Feminista de Rosa* (Rosa´s Feminist House); and *Revista Desafiando* (Challenging) from Fundación Desafío (Challenge Foundation).

† Non-commercial, often homemade or online, publication usually devoted to specialised and often unconventional subject matter.

‡ *Comadreando* translates to gossiping, chitchatting, or having a heart-to-heart chat with close friends or confidantes.The term derives from *comadre* (meaning co-mother, godmother, or very close female friend), which is also the name of a network of abortion companions. It implies a cosy, social conversation – often over coffee – that builds mutual trust and community.

¶ In Ecuador, the term “cholo” or “chola” is used to describe urbanized Indigenous people, and is part of the broader mestizaje narrative, which refers to the blending of Indigenous and European cultures. Although it was previously used as a derogatory word, the feminist collectives have reappropriated it and use it to disidentify from white feminism.

§ Refers to the exploitation of natural resources (through mines, oil exploitation, and other) within Indigenous territories without benefitting those territories, and leaving behind contamination and violence.

## Provenance statement

This article was not commissioned and has gone through external peer review.
